# Epidemiology and Cost of Peste des Petits Ruminants (PPR) Eradication in Small Ruminants in the United Arab Emirates—Disease Spread and Control Strategies Simulations

**DOI:** 10.3390/ani11092649

**Published:** 2021-09-09

**Authors:** Eihab M. Fathelrahman, Aaron Reeves, Meera S. Mohamed, Yassir M. Eltahir Ali, Adil I. El Awad, Oum-Keltoum Bensalah, Afra A. Abdalla

**Affiliations:** 1 Department of Integrative Agriculture, College of Agriculture and Veterinary Medicine, United Arab Emirates University, Al Ain P.O. Box 15551, United Arab Emirates; a.alawad@uaeu.ac.ae (A.I.E.A.); afra.abdalla@uaeu.ac.ae (A.A.A.); 2 Epidemiology Research Unit, Department of Veterinary and Animal Science, Northern Faculty, Scotland’s Rural College (SRUC), Inverness EH9 3JG, UK; Aaron.Reeves@sruc.ac.uk; 3 Epidemiology Unit, Animals Health Division, Abu Dhabi Agriculture and Food Safety Authority (ADAFSA), Abu Dhabi P.O. Box 52150, United Arab Emirates; meera.ahmed@ADAFSA.GOV.AE (M.S.M.); Yassir.Eltahir@ADAFSA.GOV.AE (Y.M.E.A.); OumKeltoum.Bensalah@ADAFSA.GOV.AE (O.-K.B.)

**Keywords:** peste des petits ruminants (PPR) disease spread, small ruminates, camels, control strategy, costs, vaccination, eradication

## Abstract

**Simple Summary:**

Peste des petits ruminants (PPR), also known as sheep and goat plague, is a highly contagious animal disease affecting small ruminants and camels. It is caused by a virus belonging to the genus Morbillivirus, family Paramixoviridae. Once newly introduced, the virus can infect up to 90 percent of an animal herd. A PPR outbreak is an emergency due to its rapid spread and high animal mortality rate. This study simulated three control strategies of PPR spread among animals in the United Arab Emirates. These strategies include implementing mass vaccination, ring vaccination and ceased vaccination strategies, combined with or without strict animal movement control simultaneously. The simulation results compared the level of the effectiveness and direct government costs for each of the three strategies. Such results aid the decision-makers in the country and globally in line with the World Animal Health Organization’s goal to eradicate the disease by 2030.

**Abstract:**

Peste des petits ruminants (PPR) is an important infectious viral disease of domestic small ruminants that threatens the food security and sustainable livelihood of farmers across Middle East, Africa, and Asia. The objective of this research is to analyze the disease’s spread and its impacts on direct government costs through conducting three simulations of different control strategies to reduce and quickly eradicate PPR from the United Arab Emirates in the near future. A Modified Animal Disease Spread Model was developed in this study to suit the conditions of the United Arab Emirates. The initial scenario represents when mass vaccination is ceased, and moderate movement restrictions are applied. The second scenario is based on mass vaccination and stamping out the disease, whereas the third simulation scenario assumes mass and ring vaccination when needed, very strict movement control, and stamping out. This study found that the third scenario is the most effective in controlling and eradicating PPR from the UAE. The outbreak duration in days was reduced by 57% and the number of infected animals by 77% when compared to the other scenarios. These results are valuable to the country’s animal health decision-makers and the government’s efforts to report to the World Animal Health Organization (OIE) regarding the progress made towards declaration of the disease’s eradication. They are also useful to other concerned entities in other Middle Eastern, North African, and Asian countries where the disease is spreading.

## 1. Background

**Figure 1:**
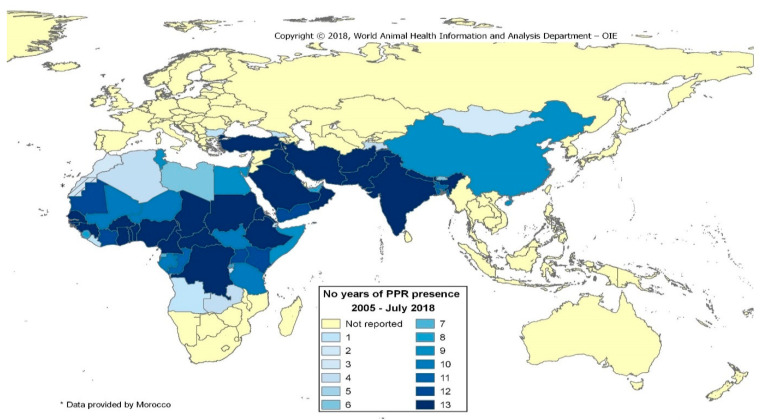
Length of PPR’s presence in years from 2005 to 2018 around the world. Source: World Animal Health Organization. https://www.oie.int/en/disease/peste-des-petits-ruminants/ accessed on 20 June 2021.

Peste des petits ruminants (PPR) is a viral disease, caused by a morbillivirus closely related to rinderpest virus, which mainly affects goats, sheep, and some wild relatives of domesticated small ruminants, as well as camels. Wild ruminants may play an important epidemiological role as a virus source for domestic small ruminants [1]. PPR was first reported in Ivory Coast in 1942. Today, more than 70 countries have confirmed PPR within their borders, and many countries are at risk of the disease being introduced. PPR is characterized by high morbidity and mortality rates of up to 90% [2]. Affected animals present with high fever, depression, along with eye and nose discharges, severe pneumonia and diarrhea. The PPR virus (PPRV) does not cross from animals to infect humans [3]. The availability of effective and safe live attenuated PPR vaccines in sheep and goats has boosted the control program in some developing countries, and currently a total of 59 countries are recognized by the OIE as being free from the disease (Figure 1). Other developing countries are currently unable to develop and apply an effective strategy to control and eradicate the PPR virus (Figure 1).

The Food and Agriculture Organization (FAO) reported that PPR affects 30 million animals across 70 countries around the world. Sixty percent of these countries are in the African continent, and the other 40% are in the Middle East and Asia. (FAO, OIE Report [3]). The disease causes annual economic losses of up to USD 2.1 billion each year. Looking beyond this figure, 300 million families are at risk of losing their livelihoods, food security, and employment opportunities. The inability of families, communities, and institutions to anticipate, absorb, or recover from PPR can compromise national and regional development efforts, and turn back the clock on decades of progress.

**Figure 2:**
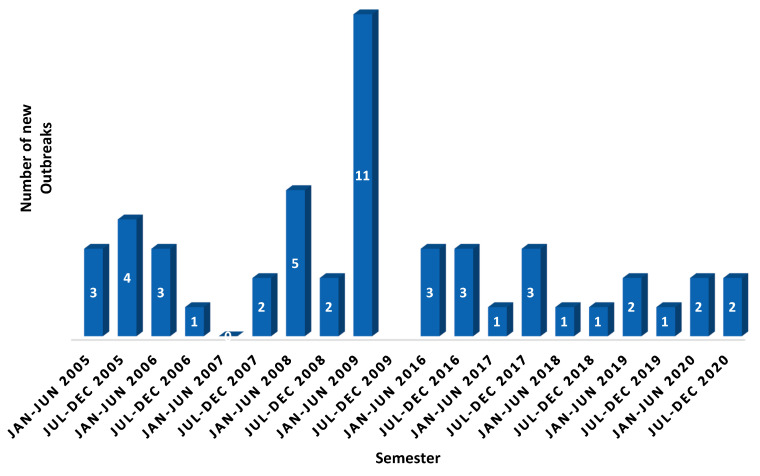
Number of PPR outbreaks from 2005 to 2020 across the UAE. Source: OIE-WAHIS. https://wahis.oie.int/#/dashboards/qd-dashboard/ accessed on 20 August 2021 [5].

**Figure 3:**
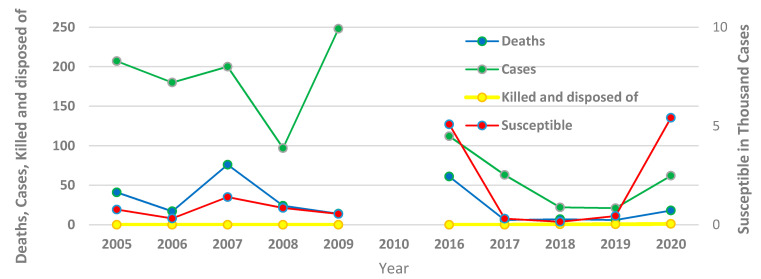
Quantitative data on PPR outbreaks from 2005 to 2020 across the United Arab Emirates (UAE). Source: OIE-WAHIS. https://wahis.oie.int/#/dashboards/qd-dashboard/ accessed on 20 August 2021 [5].

In the United Arab Emirates (UAE), PPR was first reported in wildlife in 1986. To date, a total of fifty outbreaks in both wildlife and small ruminants have been reported to the OIE. After implementing the UAE national animal health plan in 2016, which adopted a mass vaccination strategy to control and eradicate PPR, outbreaks have sharply decreased to two to three outbreaks per annum. The UAE is currently at stage 2 of the five stages of the progressive step-wise approach for the prevention and control of PPR. Despite the progress achieved in controlling PPR in the country, no simulations or trials to assess its spread and the effectiveness of different control strategies have been carried out [3,4], Figure 2 and Figure 3 (Supplementary Table).

Few studies have been reported in developing mathematical models to simulate and assess the effectiveness of various PPR control strategies. Mitchell et al. (2017) [6] noticed a lack of empirical studies on PPR. Such models provide policy-makers with a tool to develop powerful containment strategies for handling out-breaks of PPR by understanding how it spreads through herds, the uncertainty of the disease parameters, and the impact of each herd’s configuration on the disease’s spread. Therefore, the mathematical model, which can be used to configure any infectious disease, led to the finding that lowering the amount of time from the first identification of PPR in a herd to vaccination will significantly reduce the number of deaths that result from PPR [7].

Lyons et al. (2019) [8] estimated various cost components in pastoral and mixed-crop livestock systems in four PPR vaccination campaigns in Ethiopia. The cost of the overall vaccination process for mixed-crop livestock systems is approximately double the price in pastoral areas. Due to the lack of knowledge about the transmission potential of the PPRV, Fournié et al. (2017) [9] used a dynamic model of transmission and elimination of PPR in Ethiopia. The outcome was an estimation for the vaccination coverage required for elimination and the level of viral transmission in an endemic setting.

Thus, this study aimed to develop a model for PPR spread in the UAE, simulate possible control and eradication strategies, and to evaluate the effectiveness and direct government cost of such strategies. To achieve this, three scenarios of PPR spread in UAE with a corresponding different control strategy were simulated and discussed using The North American Animal Disease Spread Model (NAADSM) [10].

## 2. Global Strategy to Eradicate Peste des Petits Ruminants (PPR)

PPR can be eradicated worldwide by 2030. It can be readily and cost-effectively diagnosed, and a reliable, inexpensive and high-quality vaccine is available that confers lifelong immunity to vaccinated animals after a single dose. The virus also has a relatively short infectious phase and does not survive for long outside a host, making it an ideal candidate for a concerted eradication effort. Strengthening the capacities of national veterinary services to control and eradicate this disease will also generate wide-ranging benefits in the fight against other animal diseases [7].

**Figure 4:**
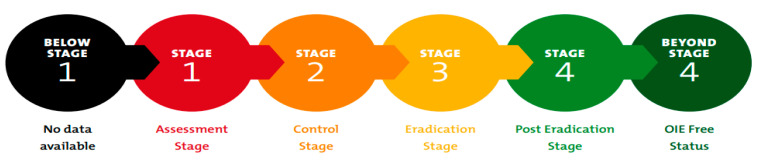
Stages of the global PPR eradication program to declare a country as being PPR-free according to the OIE.

The growing international consensus and political support for the eradication of PPR, technical feasibility, high rates of return on investment that span generations, and the proven FAO–OIE partnership in successfully eradicating transboundary animal diseases—such as rinderpest—are all strong guarantees for the success of the global PPR eradication program Figure 4. The stages of this program range from stage 1, when the epidemiological situation is assessed, to stage 4, when the country can provide evidence that there is no virus in circulation, and it is ready to apply for the OIE official status of freedom from PPR. A country is “below stage 1” if there is no epidemiological information available, and “beyond stage 4” if the OIE official status recognition has been concluded. Currently, the United Arab Emirates (UAE) shows evidence of stage 2 to stage 3 being achieved [7].

The eradication of PPR in the UAE is one of the most important goals for relevant government and non-government institutions in the country. The UAE is implementing an on-going strategy to eradicate PPR. The progress achieved by June 2021 toward the country’s PPR eradication strategy following the OIE guidelines described in the previous section of this study is assessed to be stage 3.

## 3. Objective and Simulation Scenarios

The objective of this study is to analyze the impacts (in terms reducing the epidemiological impact as well as the estimated direct government cost) of control strategies through simulations to limit and rapidly eradicate PPR from UAE.

## 4. Data and Method

This section includes a detailed description of the study data and the method applied in this study.

### 4.1. Data

Data on the population of livestock were obtained from the Abu Dhabi Agriculture and Food Safety Authority (ADAFSA) Animal Health Division. Data were collected on animals from 24,836 holdings and farms. The total population of all of the animals considered in this study was nearly 3 million heads. In this model, a cluster of animals was called a “unit”, which was the basis of the simulation. A unit had a product type, a number of animals, a point location (expressed in terms of longitude and latitude), and a disease state. All small farms had 30 animals or less of one type or a mixture of various species (sheep, goats, and camels). The majority, 14,914 farms, representing 60% of the farms in the country, harbored either sheep or goats or both sheep and goats in this study, and these were considered as one type of production due to the epidemiological similarity between the two species when it comes to PPR spread. There were 8410 small mixed sheep and goats farms, representing 34% of the total farms. The camel farms included in this study numbered 1484 farms, representing 6% of the total farms. Camels were included and assumed to be able to be infected because there is evidence of PPR outbreaks in the region [11,12]. Dairy cattle, which are mostly commercial large operations farms in the country, are found on 26 farms containing 39,750 heads of milking cows, calves, and heifers. Dairy cattle farms are part of the animal population database. However, this study does not assume any PPR infections to occur at the dairy cattle farms.

### 4.2. Methodology and Simulation Scenarios

To analyze the impacts on PPR (in terms reducing epidemiology as well as estimated direct government cost) of control strategies to eradicate it from the UAE in a timely manner, three different simulation scenarios (A, B and C) were carried out using the NAADSM as shown below:

- This scenario illustrates the case where there is no virus circulation either at the zonal or national level and the country is ready to apply for official OIE recognition of PPR freedom. This scenario represents a strategy that recognizes the initial situations when the country, here the UAE, has already controlled the disease in areas where it is highly endemic, mass vaccination is ceased (no vaccination), and moderate movement restrictions are applied.
- This scenario represents the case where efforts and the control strategy are based on mass vaccination and stamping out the disease to reduce the impact of PPR at the national level to the minimum level possible.
- The full eradication scenario represents a strategy based on mass and ring vaccination when needed (vaccinating detected herds when they occur in the vaccination ring), very strict movement controls, and stamping out the disease.

Epidemiologic modeling is a common tool used to simulate and develop possible scenarios to estimate the potential impact of outbreaks of contagious diseases, such as PPR, in populations of domesticated animals. The information generated from these scenarios is very useful and can be used by policymakers to control diseases and plan for early and on-going efforts and responses to possible outbreaks for disease management and eradication. Several spatially explicit stochastic epidemic simulation models have been developed for estimating the spread of highly contagious animal diseases and simulate outbreaks of diseases (e.g., Bates et al., 2003 [13]; Garner and Beckett, 2005 [14]). The North American Animal Disease Spread Model (NAADSM) was the adopted framework and software used in this study. The software was designed to simulate the spread and control of foreign animal diseases in a population of susceptible livestock herds (Harvey et al., 2007 [15]).

## 5. PPR Epidemiology and Cost

The following are the epidemiological features of PPR spread that were carefully modelled using NAADSM:

- The morbidity rate in susceptible populations can reach 90–100%;
- Mortality rates vary among susceptible animals, but can reach 50–100% in more severe instances;
- Both morbidity and mortality rates are lower in endemic areas and in adult animals when compared to the young;
- The latent period is considered 3 days minimum, 7 days mode, and 10 days maximum using the Beta-PERT probability function;
- The period required for herd-level natural immunity to be achieved, either through infection and recovery or vaccination, is considered to be 1460 days (4 years). Natural immunity was modeled as having a normal distribution;
- The probability of an infected animal dying from the disease is considered to be 90% because of the high mortality rate of the disease [2,16].

### 5.1. PPR Detection and Clinical Diagnosis

The probability that the owner or veterinarian will observe the death form the suspected PPR infection and report it is 100%, as the UAE government considers the disease as being mandatorily notifiable to the relevant authorities. Diagnostic testing’s sensitivity in this study was assumed be 95%. The delay in obtaining testing results was assumed to be from no delay to 3 days maximum.

### 5.2. PPR Disease States

**Figure 5:**
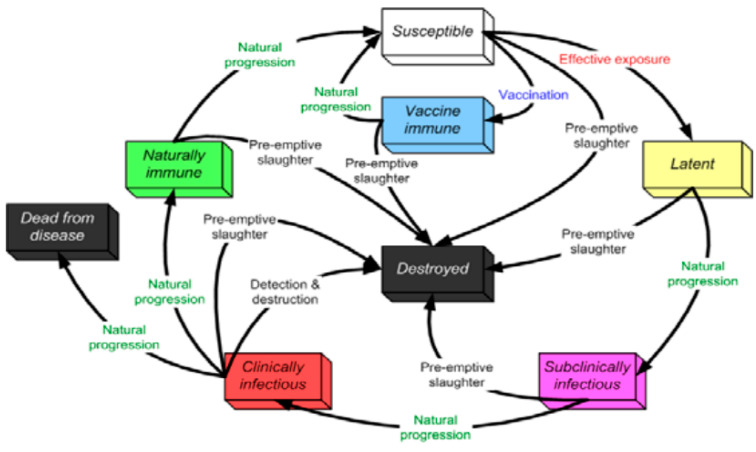
States and transitions simulated by NAADSM.

Seven discrete disease states were used in the NAADSM, as shown in Figure 5. These states are susceptible, latent, sub-clinically infectious, clinically infectious, naturally immune, vaccine immune, and dead from the disease. When a susceptible unit is infected, the natural progression is to become latent, unless a disease control action is taken. Moreover, an infected unit will proceed naturally from its latent state to a sub-clinically infectious state, unless a disease control action is taken. The disease transition will follow the natural progression, as shown in the outer loop. Implementation of any type of disease control may alter the natural disease cycle, as shown inside the loop.

### 5.3. Animals Movements and Indirect Contacts

The direct and indirect contact parameters used in this study that impact PPR spread in the UAE are summarized below (Table 1).

#### 5.3.1. Direct Contact Spread Parameters

Movement or shipment of animals among units/herds

- Mean rate of animal shipments (number of recipient units per source unit per day);
- Movement distance (probability density function per km);
- Shipping delay (probability density function per days);
- The probability of infection of the recipient unit, given exposure to an infected unit;
- Movement rate multiplier (scalar value as a function of the number of days since the first detection of the outbreak).

#### 5.3.2. Indirect Contact Spread Parameters

Indirect contacts may include the movement of people, equipment and materials, vehicles, animal products, etc., among units, and are simulated in the same manner as direct contact, except that only sub-clinically infectious and clinically infectious units, not latent units, can act as the source of infection. The parameters for indirect contact are similar to, but independent of those for direct contacts.

## 6. Peste des Petits Ruminants (PPR) Control Strategies

The NAADSM uses the following measures to control the disease: zoning and tracing, quarantine, depopulation, and vaccination parameters.

### 6.1. Zoning and Tracing

Trace out investigations by NAADSM are simulated after an infected unit is detected by zoning into two zones, surveillance low-risk zones and restricted high-risk zones. Tracing is one-level forward. The probability of successful tracing is assumed to be 80%. The maximum delay in tracing forward is assumed to be 3 days.

### 6.2. Isolation and Quarantine Parameters

In the model, units are quarantined for one or more of the following reasons: an infected unit is immediately quarantined following detection, units traced out are also quarantined, and lastly when units are selected and waiting for destruction.

### 6.3. Destruction (Depopulation) Parameters

The following are the depopulation parameters and triggers used:

- Delay to beginning a destruction program (fixed integer value per days)
- Destruction capacity (relational function: number of units that can be destroyed as a function of the number of days since the first detection of an outbreak);
- Destruction priorities (rank order of reasons for unit destruction, as described in the text);
- The radius of the destruction ring, if a ring is triggered;
- Destruction capacity is assumed to be 100 units/herd per week

### 6.4. Vaccination and Immunity Parameters

The following are the vaccination parameters and triggers used:

- Number of units that must be detected before vaccination begins (number of detected units), which is assumed to be one unit/herd detected;
- Vaccination capacity (relational function: number of units that can be vaccinated as a function of the number of days since the first detection of an outbreak). The vaccination capacity in the model is assumed to 100 units/herd per week;
- Vaccination priorities (Rank order of reasons for unit vaccination)—vaccinate detected unit when they occur in the vaccination rings;
- The radius of the vaccination ring, if a ring is triggered and other parameters, which is assumed to be 3 km.

## 7. Costs Estimation Parameters

The direct costs associated with destruction and vaccination can be calculated in the model to compare the costs of different control measures. Below are some cost input parameters used in NAADSM for determining the direct costs associated with disease control—see Appendix A on the cost parameters used to estimate direct government costs for the following items:

Parameters associated with destruction:

- Appraisal;
- Cleaning and disinfection;
- Euthanasia;
- Indemnification;
- Carcass disposal;
- Parameters associated with cost of vaccination;
- Number of animals that can be vaccinated at the baseline cost;
- The baseline cost of vaccination;
- Additional cost incurred when the number of animals vaccinated exceeds the threshold set;
- Cost of vaccination site set-up.

## 8. Peste des Petits Ruminants (PPR) Simulation Results and Discussion

**Figure 6:**
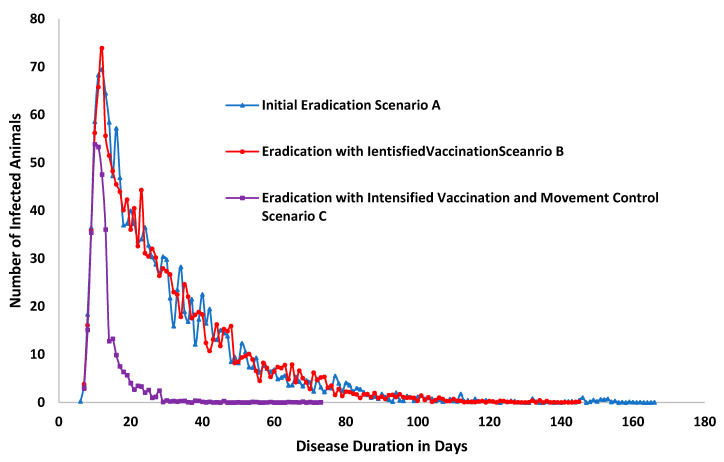
Absolute number of infected animals and outbreak duration in the three scenarios.

**Figure 7:**
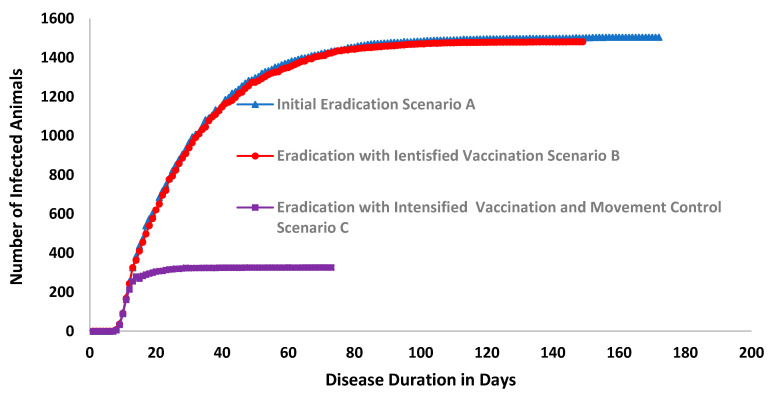
Cumulative number of infected animals and outbreak duration in the three scenarios.

Simulation results are illustrated in Figure 6 and Figure 7, and in Table 2 below. The results indicate that the outbreak durations are 171, 148, and 73 days for scenarios A, B, and C, respectively. The total cumulative number of infected animals was reported to be 1505, 1481, and 327 animals for the three scenarios A, B, and C, respectively.

The number of susceptible animals was reported to be 2,954,213, 2,954,213, and 2,954,213 animals for the three scenarios A, B, and C, respectively, while the number of latent animals was reported to be 1327, 1316, and 315 animals in the three scenarios A, B, and C, respectively. The number of animals showing subclinical signs was reported to be 887, 889, and 271 animals for the three scenarios A, B, and C, respectively. Moreover, the number of animals showing clinical signs was reported to be 690, 697, and 255 animals for the three scenarios A, B, and C, respectively.

Tracing had also been addressed as number of animals exposed to any infected herd were reported to be 2454, 2476, 227 animals of the three scenarios A, B, and C, respectively. While number of animals directly exposed that could possibly have been traced forward were reported to be 1833, 1837, 71 animals of the three scenarios A, B, and C, respectively. Total number of animals in units successfully identified by tracing (either forward or back) after direct contact 1638, 1644, 63 animals of the three scenarios A, B, and C, respectively. Number of animals in units successfully identified by tracing (either forward or back) after contact (either direct or indirect) were reported to be 2220, 2248, 208 animals of the three scenarios A, B, and C, respectively.

Regarding diagnostic testing, data showed that the number of animals subjected to diagnostic testing after a trace forward or trace back after direct contact was reported to be 1444, 1461, and 56 animals for the three scenarios A, B, and C, respectively. Meanwhile, the number of animals subjected to diagnostic testing after a trace forward or trace back after (either direct or indirect) contact was reported to be 1962, 2007, and 421 animals for the three scenarios A, B, and C, respectively. Additionally, the number of animals in the tested units with a true negative diagnostic test result was reported to be 1046, 1111, and 1 animals in the three scenarios A, B, and C, respectively.

The number of animals that are destroyed was reported to be 1384, 1370, and 321 animals in the three scenarios A, B, and C, respectively.

Niu et al. (2017) [17], based on the global effort to combat PPRV, presented a global online prediction system by adopting correlational analysis, based on collected data from 2977 cases from 2009 to 2018, and showed that PPR has a severe impact on people depending on the livestock production system as a means to generate income to reduce poverty.

Controlling PPR is essential for poverty alleviation, especially in Africa, the Middle East, and South Asia. Additionally, the model results show that the outbreaks were concentrated in the continents of Asia and Africa, and widely spreading in the Middle East region [18].

Cameron (2019) [19] found that a more sustainable option for PPR eradication could be adopting guerrilla tactics, where the primary weapon is information and understanding PPR. This tactic can be divided into four main phases—the foundation, planning, implementation and demonstration of global freedom. The author asserted that continuous real-time information in the form of guerilla tactics should be the primary tool for disease eradication, not long-term vaccination. This will also optimize the use of available sources and minimize the disruption related to managing the movement of animals from infected to uninfected areas. We herein developed a model for the disease spread of PPR, simulated possible control and eradication strategies, and evaluated the effectiveness and direct government cost of such strategies.

These results show that control strategies that intensify vaccination compared to no vaccination and with no strict and intensive animal movement controls moderately reduce the outbreak duration by 57% and the spread of PPR by 78% when compared to vaccination with stamping out (B) or ceased vaccination (A) strategies. However, an integrated and targeted eradication and control strategy that applies all possible effective measures of both triggered ring vaccination and animal movement controls reduced the disease’s outbreak duration in scenario C by 57% and the total cumulative number of infected animals by 78% compared to the initial eradication scenario A (without vaccination and lesser restriction of animals’ movement).

Scenario B address the impact of vaccination. Efficient PPR vaccines are available and can induce life-long protective immunity in vaccinated animals.

The total number of susceptible animals is nearly 3 million animals in the UAE. The simulation scenarios detailed the control strategies that can be applied against PPR, and we discuss the outcomes and perform a comparison between the three scenarios’ outcomes, as illustrated in Table 2 below. The comparison between scenario A and scenario B shows the changes due to the introduction of the triggered vaccination as the disease control strategy. Due to vaccination, the outbreak duration on average of the model’s 1000 iterations reduced from 171 days to 141 days, or by −13%. The number of infected animals slightly reduced from 1103 to 1092, or by −1%. Similarly, all other parameters regarding disease spread and the impact of the vaccination strategy showed changes that varied from 0% (no change) to −1%. This indicates that a reliance on vaccination measures alone (e.g., not accompanied by restrictions on animals’ movement) would not contribute enough to achieve PPR eradication. However, the most effective scenario, scenario C, showed a reduction in the disease’s spread or the outcome of the control strategy in the range from 57% for the disease duration in days to as high as 96% for the number of animals directly exposed that could have been traced forward. The number of animals depopulated reduced from 1384 animals to only 324 animals, or by 77% in scenario C compared to scenario A. The number of vaccinated animals was also reduced from 35,061 to 2704. These results, compared to the progress the country has achieved (reaching stage 2 of PPR eradication) as of 2021, applying PPR national eradication strategy, indicate that a successful eradication strategy may apply a very intensive and strict movement control strategy integrated with vaccination and depopulation only when such measures are triggered. Such observations are in line with the global PPR control and eradication strategy, where it is stated that prevention and control measures are a combination of different tools, which can include vaccination, improved biosecurity, animal identification, movement control, quarantine and stamping out. These individual tools are likely to be applied at different levels of intensity as an individual country moves along the pathway. (FAO OIE Global Strategy for the Control and Eradication of PPR. Paris: OIE and FAO; (2015).

## 9. Direct Government Costs Comparison of PPR Eradication Scenarios Results

Table 3 below shows a comparison of the various scenarios’ direct government cost estimates. The scenario simulation results show the direct government cost are found to be of 1.1, 1.5, and 0.2 million US Dollars in scenario A, B, and C for each to the three scenarios, respectively. The most expensive item in all three scenarios is the cost of vaccination (about 0.5 million US dollars), followed by the cost of depopulation indemnification, which is compensation provided to the farmers when their animals are depopulated (0.4 million US dollars) and the the cost of cleaning and disinfecting the depopulated farms (0.4 million US dollars). These results indicate the presence of a trade-off between applying a vaccination strategy with less strict animal movement controls versus vaccination along with more strict animal movement controls. The latter strategy, strategy C, is more effective from a government costs perspective; mass and ring vaccination, very strict movement controls, and stamping out measures will collectively lead to the faster eradication of PPR (FAO OIE Global Strategy for the Control and Eradication of PPR. Paris: OIE and FAO; (2015)).

## 10. Conclusions

The inter-regional consultative meeting on the FMD and PPR situation, labelled as an OIE/FAO GF TADs event and held in Jordan in 2014, considered PPR’s spread across the Middle East and North Africa and other regions in Asia, and pointed to vaccination as a very effective tool, but it should not be considered as the only tool, and instead should be combined with other tools, in particular the control of the movement of live animals.

The meeting recommended the preparation of specific tools, including a monitoring and evaluation tool, a post-vaccination monitoring tool, a PPR Global Research and Expertise Network (PPR-GREN) to assess the socioeconomic impact of PPR in livestock production, livelihoods and food security, and cost/benefit analysis of PPR control options used when preparing national control programs and financial project proposals [20].

The UAE aims to report the progress of its PPR control efforts to the OIE [9] in order to be recognized as a country free from PPR. The parameters used for the disease spread were localized to UAE settings as described. The total animal population considered in this study is nearly 3 million heads from 24,836 holdings and farms. The majority (60%) of the farms in the country are sheep and goat farms, mixed small farms comprise 34% of the farms, while camel farms represent 6% of the total farms. Eradication control strategies, including zoning and tracing, vaccination, movement control, and stamping out, are all effective strategies. The triggered vaccination control strategy was necessary to reach stage 4 of the global PPR control strategy. However, comparison between scenario A and B did not show high effectiveness for triggered vaccination. Movement control was found to be more effective in reducing the number of infected animals and disease duration. To move to stage 4 and beyond stage 4 (free status), it is recommended to apply triggered vaccination, movement control and stamping out animals as needed. The analysis of the various control strategies must include assessment of the costs and resources needed for successful eradication strategies. Business losses due to restricted movement control and so businesses disruption due to movement control have to be taken into consideration when the country implements disease eradication strategies. The simulation scenarios presented here are applicable to other countries in the Middle East, Africa, and Asia, where several countries are joining the global effort by the World Animal Health Organization (OIE) and its strategy to eradicate PPR by 2030.

## 11. Limitations and Future Research

The limitations of this model include modelling within-herd, local area and airborne disease spread. Furthermore, stochastic methods for unexpected low-probability high-impact shocks should be added to the modeling, accounting for the private business losses due to the movement control and restrictions. Future research may expand the modelling and control strategies considering within-herd dynamics and the reaction of market prices to the spread of PPR.

## Figures and Tables

**Table 1:** Direct and indirect contact and movement parameters.

	Unit	Scenario A	Scenario B	Scenario C
**Direct Contact: Main Base-line Contact Rate:**	Shipments of Units/Unit/Day			
Sheep and Goat Commercial Farms		0.1	0.1	0.015
Small and Goat Small Farms		0.1	0.1	0.015
Camels Farms		0.5	0.5	0.015
**Indirect Contact: Probability of Infection Transfer:**	0–1 probability			
Sheep and Goat Commercial Farms		0.5	0.5	0.25
Small and Goat Small Farms		0.5	0.5	0.25
Camel Farms		0.25	0.25	0.25
**Distance Distribution of Recipients Units:**	Beta-PERT Distribution (Minimum. Mode, and Maximum) in Kilometers			
Sheep and Goat Commercial Farms		0, 20, 400	0, 20, 400	0, 20, 400
Small and Goat Small Farms		0, 20, 400	0, 20, 400	0, 20, 400
Camel Farms		0, 20, 400	0, 20, 400	0, 20, 400

**Table 2:** Simulation scenarios’ outcomes/results.

Outcome over the Iterations (Number of Animals in all Units or Duration in Days)	Initial Eradication Scenario (Scenario A)	Eradication with Intensified Vaccination Scenario (Scenarios B)	Percentage Change in Scenario B from Initial Scenario A (Scenario B—Scenario A)/Scenario A	Complete Eradication (Intensified Vaccination and Movement Control (Scenario C)	Percentage Change in Scenario C from Initial Scenario A (Scenario C—Scenario A)/Scenario A
Number of susceptible animals	2,954,213	2,954,213	-	2,954,213	-
Number of latent animals	1327	1316	−1%	315	−76%
Number of animals showing subclinical signs	887	889	0%	271	−69%
Number of animals showing clinical signs	690	697	1%	255	−63%
Number of animals that are destroyed	1384	1370	−1%	321	−77%
Number of animals becoming infected (not including initially infected units)	1103	1092	−1%	170	−85%
Number of animals exposed to an infected animal	2454	2476	1%	227	−91%
Number of animals directly exposed that could possibly have been traced forward	1833	1837	0%	71	−96%
Total number of animals in units successfully identified by tracing (either forward or back) after direct contact	1638	1644	0%	63	−96%
Number of animals in units successfully identified by tracing (either forward or back) after contact (either direct or indirect)	2220	2248	1%	208	−91%
Number of animals in unique units detected for any reason	1384	1370	−1%	321	−77%
Number of animals subjected to diagnostic testing after a trace forward or trace back after direct contact	1444	1461	1%	56	−96%
Number of animals subjected to diagnostic testing after a trace forward or trace back after (either direct or indirect contact) contact	1962	2007	2%	421	−79%
Number of animals in tested units with a true negative diagnostic test result	1046	1111	6%	1	−100%
Total number of animals vaccinated over the iterations.	-	35,061	100%	2704	−92%
Duration of outbreak in days	171	148	−13%	73	−57%

**Table 3:** Itemized direct government costs of eradication scenarios in Dirhams.

Cost Item	Initial Eradication Scenario	Eradication with Intensified Vaccination Scenario	Percentage Change from Initial Scenario (Scenario B—Scenario A)/Scenario A	Complete Eradication (Intensified Vaccination and Movement Control) Scenario	Percentage Change from Vaccination Triggered Ring Scenario (Scenario C—Scenario B)/Scenario B
Depopulation Appraisal	2554	2603	2%	399	−85%
Depopulation Cleaning and Disinfection	434,677	442,157	2%	66,218	−85%
Depopulation Euthanasia	99,414	98,411	−1%	23,003	−77%
Depopulation Indemnification	466,200	432,833	−7%	83,323	−81%
Depopulation Disposal	18,271	17,563	−4%	3755	−79%
Depopulation Subtotal	1,021,116	993,568	−3%	176,699	−82%
Vaccination Setup	n/a	424,381	100%	31,505	−93%
Vaccination	n/a	100,160	100%	7284	−93%
Vaccination Subtotal	n/a	524,542	100%	38,789	−93%
Total Costs	1,021,116	1,518,110	49%	215,488	−86%

## Data Availability

Not applicable.

## Supplementary Materials

The following are available online: https://www.mdpi.com/article/10.3390/ani11092649/s1.

## Appendix A

**Table A1:** Government direct cost parameters in US dollars.

A- Destruction and Cleaning and Disinfection (C&D):		Unit/Animal	Camels	Sheep and Goats	Small Ruminants Farms
	Appraisal	Unit	1183	29.7	19.71
	Cleaning and Disinfection	Unit	217,983	779.49	591.3
	Indemnification	Animal	1232	168.48	98.55
	Euthanasia	Animal	72	71.82	71.82
	Caracas Disposal	Animal	30	9.99	9.99
B- Vaccination:					
	Site Set-up	Unit	11,517	533.25	492.75
	Baseline Vaccination	Animal	3	1.89	1.08
	Number of Animals Before Vac. Cost Increases	Number of Heads	2700	135	135
	Additional Cost beyond Threshold	Animal	1	1.08	1.08
C- Zoning:					
	High Risk Zone	Animal	79	23.76	19.71
	Low Risk Zone	Animal	59	9.99	9.99
